# Understanding the Molecular Interactions Between Pandan Pigment and Food Components for Enhanced Thermal Stability

**DOI:** 10.3390/foods13213361

**Published:** 2024-10-23

**Authors:** Junxia Chen, Chunhe Gu, Mengrui Wang, Ziqing Chang, Junping Zhou, Mingzhe Yue, Fei Liu, Zhen Feng

**Affiliations:** 1 Key Laboratory of Dairy Science, Ministry of Education, College of Food Science, Northeast Agricultural University, Harbin 150030, China; chenjx202202@163.com (J.C.); wangmengrui1029@126.com (M.W.); 18830074160@163.com (Z.C.); 18434010809@163.com (J.Z.); y18204436985@163.com (M.Y.); 2 Spice and Beverage Research Institute, Chinese Academy of Tropical Agricultural Sciences, Wanning 571533, China; guchunhe688@163.com

**Keywords:** pandan pigment, sugar, protein, interaction, stability

## Abstract

Pandan pigment (*Pandanus amaryllifolius*) is widely used as a natural food coloring and flavoring agent. However, its application in food is limited because of its susceptibility to thermal degradation during food processing, which affects both pigment stability and color. Despite its growing use, there is limited research on how common food ingredients can mitigate this degradation. This study addresses this gap by exploring the effects of sucrose, lactose, rice starch, whey protein, and soy protein isolate on the thermal and color stability of pandan pigment under various heating conditions (65 °C, 95 °C, 115 °C, and 121 °C for 15 min). Spectroscopic techniques (UV–visible, infrared, and fluorescence) and laser confocal microscopy were used to elucidate the molecular interactions. The results revealed that rice starch provided the strongest protection, followed by whey protein, soy protein isolate, lactose, and sucrose, although the protective effects decreased at higher temperatures. These findings offer new insights into the use of sugars and proteins to increase the thermal stability of natural pigments in food applications.

## 1. Introduction

*Pandanus amaryllifolius*, commonly known as pandan, belongs to the Pandanaceous family and is highly valued for its distinctive aroma [1]. Its fragrant leaves, which are widely cultivated in Southeast Asia, are rich in chlorophyll, aromatic compounds, carotenoids (such as β-carotene and lutein), and lipophilic antioxidants [2]. Pandan leaves are extensively used as natural colorants and flavoring agents in traditional foods and beverages [3]. However, despite its natural origin and health benefits, the industrial application of the pandan pigment remains limited because of its poor stability during heat processing, which compromises both color and bioactivity [4].

The primary coloring agent in pandan leaves, chlorophyll, is highly sensitive to external factors such as heat, oxygen, light, and acidity, leading to rapid degradation [5]. This instability results in undesirable color changes and reduces functional properties, posing challenges for the food industry. While pandans are commonly used in fresh or processed forms across a variety of products—ranging from baked goods and desserts to teas and traditional dishes such as ‘nasi lemak’ [6], their expansion into heat-treated foods, including dairy and plant-based products, is hindered by their vulnerability to thermal degradation.

To address these challenges, previous studies have demonstrated that certain food ingredients, such as sugars and proteins, can increase the stability of natural pigments. For example, sucrose stabilizes β-carotene microcapsules [7], lactose protects betacyanin [8], and starches form strong hydrogen bonds with capsanthin to improve their coloring properties [9]. Similarly, whey protein and soy protein isolate improve the stability and bioactivity of pigments such as anthocyanins and lutein through hydrophobic, electrostatic, and hydrogen-bonding interactions [10]. Despite these advances, few studies have investigated the interaction mechanisms of these substances on pandan pigment under common heat treatment conditions. This study aims to fill that gap by systematically investigating the thermal and color stability of pandan in the presence of sucrose, lactose, rice starch, whey protein, and soy protein isolate. Advanced spectroscopic techniques, including UV–visible spectroscopy, infrared spectroscopy, fluorescence spectroscopy, and confocal laser microscopy, can be used to elucidate the interaction mechanisms between the pandan pigment and these substances. This research is expected to provide novel insights into optimizing the stability of pandan pigment for broader food applications.

## 2. Materials and Methods

### 2.1. Extraction of Pandan Pigment

The extraction method for the pandan pigment followed the procedure described by Sayoga et al. [11], with slight modifications. The freeze-dried pandan (Pandanus amaryllifolius) powder was mixed with absolute ethanol at a ratio of 1:15 (*w*/*v*) and ultrasonicated at 37 kHz for 30 min. The mixture was then centrifuged (8000× *g*, 15 min, 4 °C), and the supernatant was collected for further analysis.

### 2.2. Determination of Pandan Chlorophyll Content

The content was quantified following the method described by Li et al. [12], with slight modifications. The diluted samples were dispensed into a 96-well plate at 200 μL per sample. The absorbance was measured at 645 and 663 nm via a microplate reader (Synergy H1, Bio Tek, Winooski, VT, USA). All procedures were conducted in darkness.

(1) $$Pandan\;Chlorophyll\;concentration\;(mg⁄L)=8.04\times A_{663}+20.29\times A_{645}$$

where A_663_ and A_645_ are the absorbances at 663 and 645 nm, respectively.

### 2.3. Preparation of the Pandan Extract and Stabilizing Agent

Ethanol extracts of the pandan pigment at a concentration of 33.531 mg/mL were mixed via a magnetic stirrer at room temperature at a 1:1 (*v*/*v*) ratio with 0%, 1%, 3%, 5% and 7% sucrose, lactose, and rice starch, and 0%, 2%, 4%, 6%, and 8% whey protein and soy protein isolate.

### 2.4. Color Stability of Mixture Under Various Thermal Conditions

The mixtures of the pandan pigment with sucrose, lactose, rice starch, whey protein, and soy protein isolate were heated at 65 °C for 15 min, 95 °C for 15 min, 115 °C for 15 min, and 121 °C for 15 min. After cooling to 25 °C, the pandan chlorophyll content was determined to characterize the thermal stability. Color evaluation was performed via a colorimeter (WF32-16, Shenzhen Wave Optoelectronics Technology Co., Ltd., Shenzhen, China) following the method described by Zhang et al. [13].

### 2.5. Determination of Ultraviolet–Visible (UV–Vis) Absorption Spectra

The absorption spectra of the mixtures of the pandan pigment with sucrose, lactose, rice starch, whey protein, and soy protein isolate were recorded via a UV–vis spectrophotometer [14].

### 2.6. Determination of Fluorescence Spectra

The fluorescence spectra of the mixtures of the pandan pigment with sucrose, lactose, rice starch, whey protein, and soy protein isolate were recorded via an F-4600 fluorescence spectrophotometer (F-4600 FL spectrophotometer, HITACHI, Tokyo, Japan) at room temperature. The scan covered fluorescence emission wavelengths from 600 nm to 750 nm, with excitation at 393 nm.

### 2.7. Confocal Laser Scanning Microscopy (CLSM)

The microscopic morphology of the mixtures of the pandan pigment with sucrose, lactose, rice starch, whey protein, and soy protein isolate was visualized via confocal laser scanning microscopy (CLSM) (Fv10i, OLYMPUS, Tokyo, Japan) to determine the distribution of pigments within the mixtures, and the mixtures were excited via a laser at 488 nm [15].

### 2.8. Fourier Transform Infrared Spectroscopy (FTIR)

The lyophilized sample was finely ground and mixed with an appropriate amount of KBr. The mixture was then pressed into thin pellets, and the spectra were recorded via an FTIR spectrometer (Nicolet 6700, Thermo Fisher, Waltham, MA, USA) in the range of 400–4000 cm^−1^.

### 2.9. Statistical Analysis

Each experiment was performed in triplicate and repeated three times independently. Two-way analysis of variance (ANOVA) and significant difference tests were performed via SPSS Statistics 27, and the results are presented as the means ± SDs. The mean differences were considered significant (*p* < 0.05). Graphs were generated via the Origin Lab 2018 software (Origin Lab Corporation, Northampton, MS, USA).

## 3. Results

### 3.1. Analysis of Thermal Stability

**Figure 1:**
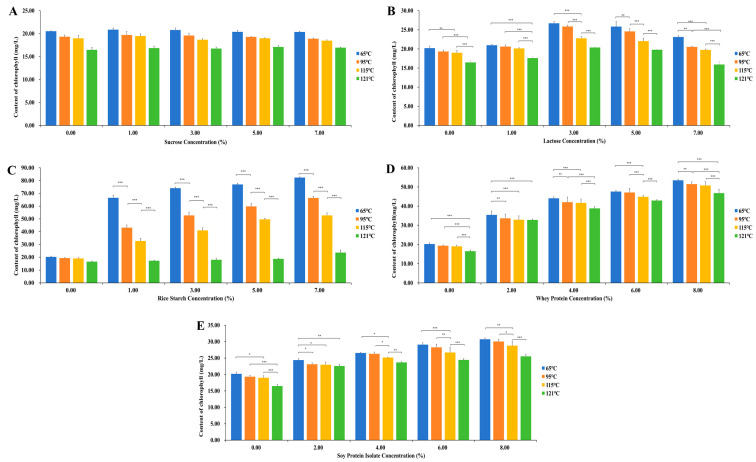
Effects of different concentrations of sucrose, lactose, rice starch, whey protein, and soy protein isolate under various heating temperatures on the thermal stability of the pandan pigment. (**A**) Pandan pigment and sucrose; (**B**) pandan pigment and lactose; (**C**) pandan pigment and rice starch; (**D**) pandan pigment and whey protein; (**E**) pandan pigment and soy protein isolate. “*”, “**”, and “***” represent significance levels of 0.05, 0.01, and 0.001, respectively. “A” The interaction between temperature and concentration is not significant, so no significance is indicated. The error bars represent the standard deviation.

Figure 1 shows the effects of different concentrations of sucrose, lactose, rice starch, whey protein, and soy protein isolate on the chlorophyll content of pandan at different temperatures. The results indicate that the interaction between temperature and concentration significantly influences the chlorophyll content, with varying protective effects observed for different additives. As shown in Figure 1A, in the sucrose–pandan mixture, the interaction effect between temperature and concentration was not significant (*p* > 0.05). However, both the sucrose concentration and heating temperature significantly affected the chlorophyll content of pandan (*p* < 0.05). At 65 °C, the pandan chlorophyll content remained relatively stable at relatively low sucrose concentrations (from 20.53 to 20.36). The pandan chlorophyll content in response to 1% sucrose (20.87) was slightly greater than that in response to 0% sucrose (20.53), suggesting that a small amount of sucrose may have a mild protective effect. At relatively high sucrose concentrations (3%, 5%, and 7%), the pandan chlorophyll content decreased as the sucrose concentration increased. This trend was also observed at other temperatures. Temperature generally has a negative effect on the pandan chlorophyll content, and regardless of the sucrose concentration, as the temperature increases, the pandan chlorophyll content decreases. At 121 °C, the chlorophyll content significantly decreased at all concentrations. In conclusion, at lower temperatures (65 °C), 1% sucrose provides better protection for pandan chlorophyll, whereas at higher temperatures, the protective effect of sucrose gradually diminishes, and increasing the sucrose concentration does not significantly improve the stability of pandan chlorophyll.

Figure 1B–E clearly show that for mixtures of the pandan pigment with lactose, rice starch, whey protein, and soy protein isolate, the interaction between temperature and concentration was significant (*p* < 0.05). As the temperature increased, the protective effect of lactose gradually weakened. At 65 °C, all concentrations of lactose maintained relatively high chlorophyll contents, especially at 3% and 5%, indicating the highest stability (26.70 and 25.83, respectively). At higher temperatures (115 °C and 121 °C), the chlorophyll content at all concentrations significantly decreased, with lactose decreasing from 16.47 to 15.93 at 121 °C. The protective effect of lactose is limited and cannot fully prevent degradation at high temperatures. As the temperature increased, the pandan chlorophyll content decreased compared with that at 65 °C, but the protective effects of rice starch, whey protein, and soy protein isolate remained significant, especially at relatively high concentrations, which effectively inhibited chlorophyll degradation. At 121 °C, the chlorophyll content significantly decreased at all concentrations, but at 7% rice starch, the chlorophyll content (23.61) remained relatively high, and at 8% whey protein, the chlorophyll content remained at 46.80, indicating a marked protective effect compared with other concentrations. Compared with other temperatures, the chlorophyll content of 8% soybean protein isolate at 121 °C was 25.53, which was lower than that at other temperatures, but significantly higher than that at the same temperature, indicating that 8% soybean protein isolate had the best protective effect.

In conclusion, at lower temperatures (65 °C), the protective effect of various additives (such as rice starch, whey protein, and soy protein isolate) was most significant, particularly at higher concentrations, which effectively enhanced the stability of pandan chlorophyll. As the temperature increased, pandan chlorophyll degradation accelerated significantly, but high concentrations of whey protein, rice starch, and soy protein isolate still slowed this process to some extent, especially at 95 °C and 115 °C. At extremely high temperatures (121 °C), although the protective effects of all the additives were somewhat weakened, certain additives (such as whey protein and soy protein isolate) still demonstrated strong protective effects at relatively high concentrations.

### 3.2. Analysis of Color Stability

Table 1 and Table 2 present the lightness (L*), redness (a*), and yellowness (b*) of the pandan pigment at various temperatures and with different concentrations of sucrose, lactose, rice starch, whey protein, and soy protein isolate.

Table 1 shows the effects of different concentrations of sucrose, lactose, and rice starch (three additives) on the stability of pandan pigment (measured by L*, a*, and b* values) at different temperatures (65 °C, 95 °C, 115 °C, and 121 °C). L* represents the brightness of the color [16], higher values indicate brighter colors, and lower values indicate darker colors. At 65 °C, the effects of different concentrations of sucrose, lactose, and rice starch on the L* values of the pandan pigment were not significant (*p* > 0.05). However, the effect of 1% sucrose was significant (16.56), whereas the other concentrations had no notable effects. As the temperature increased to 95 °C, the effects of sucrose and lactose became more pronounced, with 1% sucrose and 3% lactose resulting in lower L* values. At 121 °C, rice starch, particularly at relatively high concentrations (5% and 7%), maintained relatively high L* values, whereas sucrose and lactose at relatively high concentrations resulted in relatively low L* values.

a* represents the red–green component, with negative values indicating green and positive values indicating red. At 65 °C, the concentration of sucrose had no significant effect on the a* value of the pandan pigment (*p* > 0.05), but the concentrations of lactose and rice starch had a significant effect on the a* value (*p* < 0.05), with all values remaining within the negative range, indicating minimal color changes. As the temperature increased, the effect of sucrose concentration on a* became more apparent, with a slight increase in a* (from −2.15 to −2.05 for 1% sucrose), suggesting that higher sucrose concentrations may reduce the greenness of the pandan pigment. At 95 °C, lactose and rice starch concentrations between 1% and 5% had a significant effect on a* (*p* < 0.05), with lactose at 3% and 5% resulting in lower a* values and rice starch at 7% resulting in an a* value of −3.39, indicating stronger protection and a greener color.

b* represents the yellow–blue component, with positive values indicating yellow and negative values indicating blue. The effects of different concentrations of sucrose, lactose, and rice starch on the b* value of the pandan pigment were not significant (*p* > 0.05) at 65 °C. Rice starch, especially at high concentrations (5% and 7%), provided the strongest protection against the pandan pigment at all temperatures. As the temperature increased, the changes in the L*, a*, and b* values remained small, indicating better stability.

In conclusion, rice starch, particularly at high concentrations (5% and 7%), provided the strongest protection for the pandan pigment at all temperatures, resulting in minimal changes in the L*, a*, and b* values. Lactose performed better at low temperatures, especially at 1% and 3% concentrations, but its protective effect weakened at higher temperatures. Sucrose showed relatively consistent results across concentrations, with better protection at low concentrations (1%) but poor performance at higher temperatures.

Table 2 presents the effects of different concentrations of whey protein and soy protein isolate on the L*, a*, and b* values of pandan pigment at different heating temperatures (65 °C, 95 °C, 115 °C, and 121 °C). At 65 °C, the effects of whey protein and soy protein isolate on the L* value were minimal, but as the protein concentration increased, the L* value decreased. At 95 °C, the L* value for whey protein at a 2% concentration decreased to 15.17, whereas that of the soy protein isolate was 16.12. Although both values decreased, the L* value of the soy protein isolate remained relatively high at relatively high concentrations. Compared with whey protein, the soy protein isolate maintained a more stable brightness across concentrations. At 115 °C, the effect of whey protein became more pronounced, with the L* value at the 2% concentration decreasing to 15.16, whereas the L* value of the soy protein isolate at the 2% concentration was 15.13, maintaining a higher brightness. As the concentration increased, the L* value of whey protein gradually decreased (14.27 at 6%), whereas the soy protein isolate was more stable (15.95 at 6%). At 121 °C, the effect of whey protein on the L* value became even more significant, with a drastic decrease to 14.00 at a concentration of 6%. The L* value of the soy protein isolate decreased slightly to 15.37 at 8% but remained more stable than that of the whey protein.

At 65 °C, both whey protein and soy protein isolate had relatively low a* values, maintaining a stable greenish hue. At 95 °C, the a* value decreased significantly, with whey protein showing a stronger trend toward greenness as the concentration increased (−4.27 at 2% and −4.89 at 8%). The soy protein isolate showed similar trends, although the changes were less pronounced (−3.82 at 2% and −4.21 at 8%). At 115 °C, the a* value continued to decrease, with whey protein showing a more pronounced green shift at high concentrations (−4.29 at 8%), whereas soy protein isolate remained relatively stable (4.05 at 8%).

The effects of whey protein and soy protein isolate on the b* value of the pandan pigment did not show any consistent patterns with respect to temperature or concentration, suggesting that their effects on the color of the pandan pigment were unstable in combination with temperature and concentration. Overall, increased temperature caused a decrease in the L* values, leading to darker colors, and the b* values also decreased. However, whey protein showed a more pronounced shift towards greenness as the concentration increased, especially at high temperatures, whereas soy protein isolate presented relatively stable red–green components. Whey protein caused a slight decrease in b* values at high temperatures, whereas the yellow component (b*) of soy protein isolate remained more stable.

### 3.3. UV–Vis Absorption Spectroscopy Analysis

**Figure 2:**
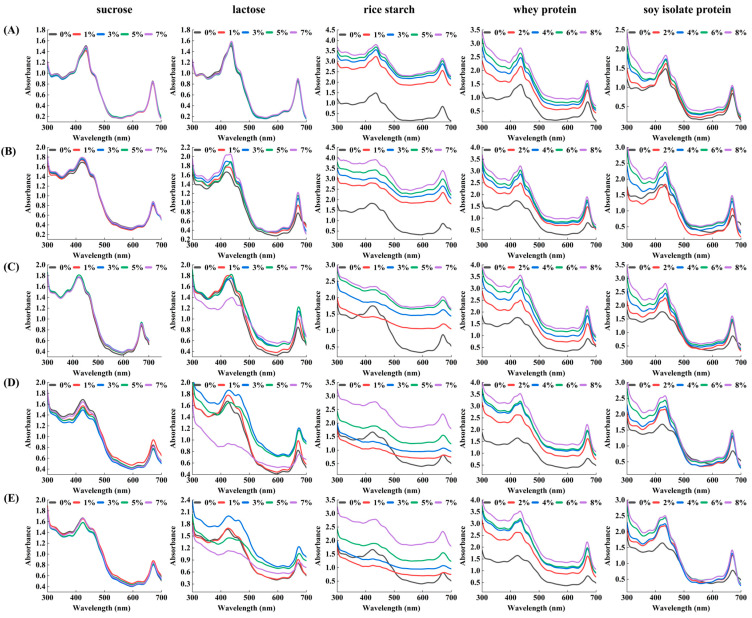
UV–visible absorption spectra (300–700 nm) of pandan pigment solutions enriched with different concentrations of sucrose, lactose, rice starch, whey protein, and soy protein isolate before and after heating. (**A**) Before heating; (**B**) heating at 65 °C for 15 min, (**C**) at 95 °C for 15 min, (**D**) at 115 °C for 15 min, and (**E**) at 121 °C for 15 min.

The spectral characteristics of the pandan pigment are shown in Figure 2, which reveals that the pandan pigment has two major absorption peaks, at approximately 425 and 670 nm. The specific structural properties of the porphyrin pigments considerably affect the UV–vis absorption spectra.

Before heating, the pandan pigment solutions containing various concentrations of sucrose, lactose, rice starch, whey protein, and soy protein isolate presented absorption spectra similar to those of the pure pandan pigment. However, a redshift towards a longer wavelength region at 425 nm was observed after these substances were bound. Compared with that of the pandan pigment alone, the absorbance of the pandan pigment increased in the presence of lactose, rice starch, whey protein, and soy protein isolate at different concentrations. This increase was significantly greater with higher concentrations of these additives, which was attributed to interactions between the chromophores in the pandan pigment and the auxochromes (-OH, -NH_2_, -SH, etc.) in the additives. Sucrose, on the other hand, did not significantly shift the maximum absorption wavelength of the pandan pigment [17], although the type and concentration of sugar caused varying degrees of hypochromic and hyperchromic effects on the pandan pigment.

The UV–vis spectra of the pandan pigment enriched with different concentrations of sucrose, lactose, rice starch, whey protein, and soy protein isolate after heat treatment are shown in Figure 2B–E. The peak shape of the absorption spectrum of the pandan pigment changed with the addition of different concentrations of sucrose, lactose, rice starch, whey protein, and soy protein isolate, and the maximum peak positions slightly shifted.

### 3.4. Fluorescence Spectrum Analysis

**Figure 3:**
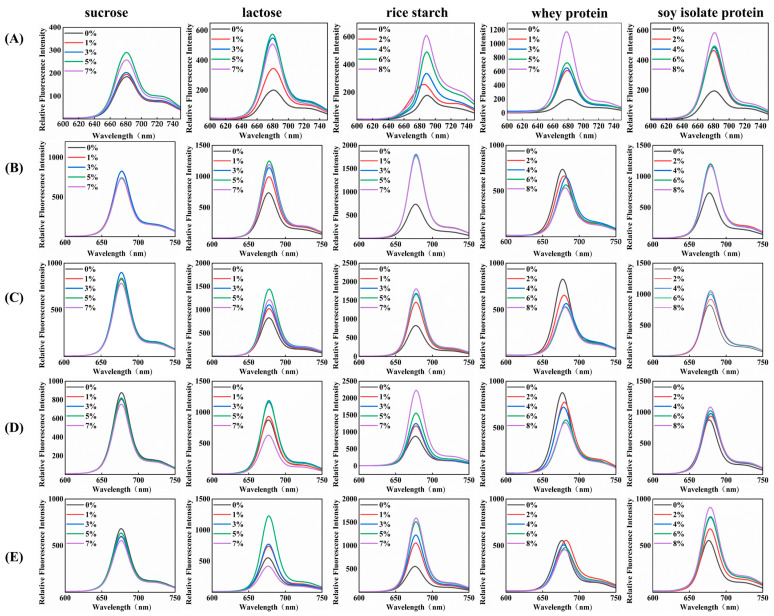
The fluorescence emission spectra of pandan pigment solutions enriched with different concentrations of sucrose, lactose, rice starch, whey protein, and soy protein isolate before and after heating. (**A**) Before heating; (**B**) heating at 65 °C for 15 min, (**C**) 95 °C for 15 min, (**D**) 115 °C for 15 min, and (**E**) 121 °C for 15 min.

Figure 3 shows the fluorescence emission spectra of pandan pigment with various concentrations of sucrose, lactose, rice starch, whey protein, and soy protein isolate before and after heat treatment. Before heating, the emission spectra of the pandan pigment solution and the additives exhibited a strong peak at 680 nm, characteristic of chlorophyll. The fluorescence intensity of the pigment mainly depends on the number of porphyrin rings. The characteristic fluorescence emission of the porphyrin ring increased with the addition of different concentrations of sucrose, lactose, rice starch, whey protein, and soy protein isolate. As shown in Figure 3, with increasing temperature, the maximum absorption wavelength of the pure pandan pigment group slightly blueshifted (680–676 nm), and its fluorescence intensity increased (195–877 A.u.). However, when the temperature reached 121 °C, the fluorescence intensity decreased (555 A.u.).

Before heating, the addition of sucrose, lactose, rice starch, whey protein, and soy protein isolate led to an increase in the fluorescence intensity of the pandan pigment. Sucrose did not alter the maximum absorption wavelength of pandan pigment at any temperature. Lactose and rice starch caused a blue shift at low temperature (before heating) but induced a red shift at higher temperatures (65 °C, 95 °C, 115 °C, 121 °C). The addition of whey protein and soy protein isolate consistently led to a red shift in the maximum absorption wavelength, with the degree of red shift increasing as the temperature rose. Although the addition of sucrose slightly increased the fluorescence intensity, it did not follow a regular concentration-dependent trend, and the absorption wavelength remained unchanged. At 65 °C, lactose and whey protein were added, which caused a significant concentration-dependent change in fluorescence intensity. At 65 °C, the whey protein–pandan pigment mixture redshifted (677–681 nm) as the whey protein concentration increased, and its intensity decreased (877–554 A.u.), resulting in fluorescence quenching. The fluorescence intensity of the sucrose–pandan pigment at 115 °C and 121 °C was lower than that of the pure pandan pigment. As the temperature increased, the maximum fluorescence intensity of the pandan pigment mixtures containing sucrose, lactose, rice starch, and soy protein isolate first increased but then decreased. Although the fluorescence intensity of all the pandan pigment mixtures reached its lowest point at 121 °C, the fluorescence intensity of the rice starch and soy protein isolate groups remained greater than that of the other groups (1593 and 911 A.U., respectively).

### 3.5. Laser Confocal Scanning Microscopy (CLSM) Analysis

**Figure 4:**
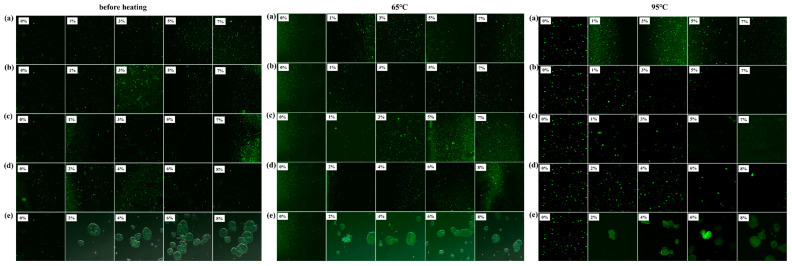

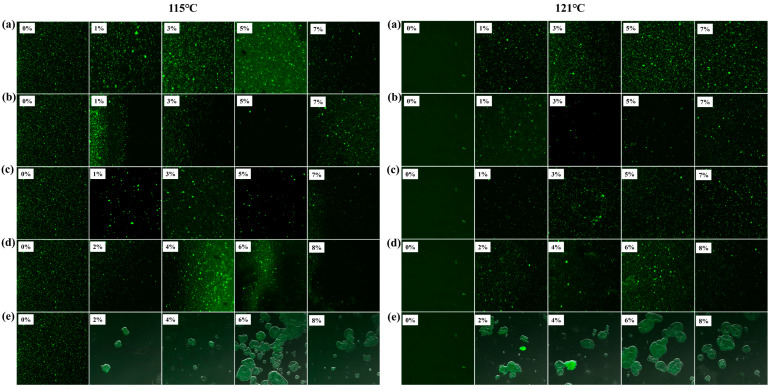
Confocal laser scanning microscopy images of pandan pigment solutions enriched with different concentrations of (**a**) sucrose, (**b**) lactose, (**c**) rice starch, (**d**) whey protein, and (**e**) soy protein isolate before and after heating at 65 °C for 15 min, 95 °C for 15 min, 15 °C for 15 min, and 121 °C for 15 min.

The images were captured via laser confocal scanning microscopy (CLSM) to observe the morphology of the pandan pigment and its mixtures with sucrose, lactose, rice starch, whey protein, and soy protein isolate both before and after preheating treatment (Figure 4). Pandan pigment, a natural fluorescent pigment, emits green fluorescence (false color) under specific laser wavelengths [18]. As shown in Figure 4, compared with those before heating, the fluorescence spots of the pure pandan pigment group gradually increased at 65 °C, 95 °C, and 115 °C but almost disappeared at 121 °C. Compared with those of the pure pandan pigment group, the addition of sucrose, lactose, rice starch, whey protein, and soy protein isolate increased the number of fluorescence spots. The status shown in Figure 4 reveals that, except for the soy protein isolate, which forms larger aggregates with the pandan pigment, all other pandan pigment mixtures are small particles. The addition of soy protein isolate caused a significant change in the fluorescence distribution of the pandan pigment, with some pigments being encapsulated within the soy protein isolate. Overall, the different temperature and concentration treatments did not affect the morphology of the pandan pigment mixtures with sucrose, lactose, rice starch, whey protein, or soy protein isolate.

### 3.6. Fourier Transform Infrared (FTIR) Spectrum Analysis

**Figure 5:**
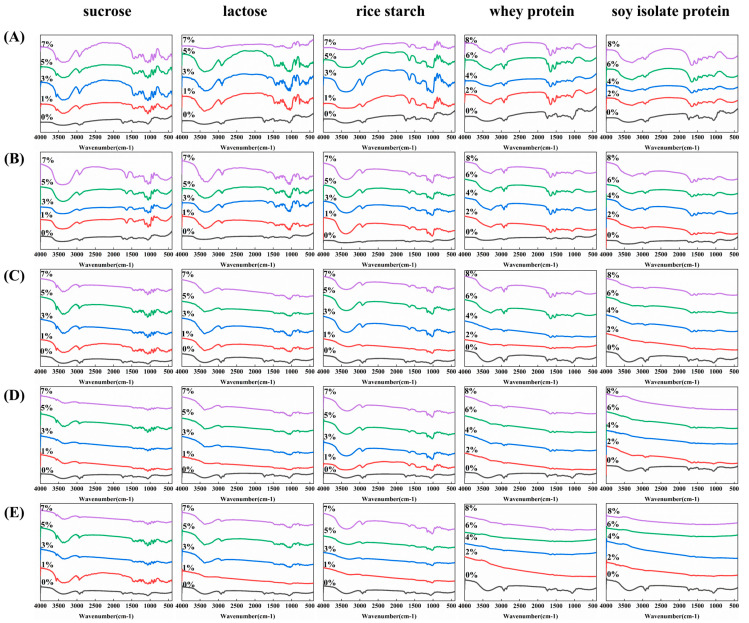
FTIR spectra of pandan pigment solutions enriched with different concentrations of sucrose, lactose, rice starch, whey protein, and soy protein isolate before and after heating. (**A**) Before heating; (**B**) heating at 65 °C for 15 min, (**C**) 95 °C for 15 min, (**D**) 115 °C for 15 min, and (**E**) 121 °C for 15 min.

The functional groups in the mixtures of the pandan pigment with sucrose, lactose, rice starch, and soy protein isolate were characterized via FTIR spectroscopy, as shown in Figure 5.

The characteristic bands of the pandan pigment appeared at 3389 cm⁻^1^ (O-H stretching), 2924 cm⁻^1^ (C-H stretching in phytol), 2862 cm⁻^1^ (asymmetric and symmetric CH₂ and CH_3_ stretching), 1737 cm⁻^1^ (C=O stretching in the methyl ester group), 1634 cm⁻^1^ (C=C skeletal vibration), 1561 cm⁻^1^ (C=N skeletal vibration), 1451 cm⁻^1^ (CH stretching), and 1384 cm⁻^1^ (C-N stretching) before heating [19]. With increasing heating temperature, the hydroxyl absorption peak of the pigment gradually intensified, reaching a maximum at 115 °C after 15 min.

Compared with that of the pure pandan pigment, the O-H absorption peak of the sucrose–pandan pigment complex shifted to a lower wavenumber (from 3399 cm⁻^1^ to 3387 cm⁻^1^), indicating hydrogen bonding between sucrose and the pandan pigment. The characteristic peak at 2862 cm⁻^1^ disappeared when the pandan pigment bound to sucrose, and the peak position shifted from 2924 cm⁻^1^ in the pandan pigment to 2931 cm⁻^1^ in the sucrose–pandan pigment complex. The main region between 1550 and 1800 cm⁻^1^, associated with the carbonyl (C=O) stretching vibration, shifted with increasing sucrose concentration. These changes in peak positions are attributed to the formation of hydrophobic interactions between sucrose and the pandan pigment. The absorption peak of the sucrose–pandan pigment mixture reached a maximum when the sucrose concentration was 3% before heating.

The infrared spectrum of the lactose–pandan pigment mixture exhibited a broad band in the 3000–3600 cm⁻^1^ region, with a prominent peak at 3382 cm⁻^1^ and additional peaks at 3346 cm⁻^1^ on the shoulders. The peaks at 1737 cm⁻^1^ and 2853 cm⁻^1^ corresponding to the pandan pigment were diminished in the lactose–pandan pigment mixture [20]. As the lactose concentration increased, a blueshift (from 3389 to 3372 cm⁻^1^) of the O-H absorption peak and a redshift (from 1060 to 1067 cm⁻^1^) were observed in the lactose–pandan pigment mixture. These results indicate that the interactions between lactose and the pandan pigment occur through hydrophobic interactions and hydrogen bonding. The absorption peak of the lactose–pandan pigment mixture reached a maximum when the lactose concentration was 1% before heating.

In the presence of rice starch, the intensity of the absorption peak in the rice starch–pigment mixture increased, and the peak at 3389 cm⁻^1^ (O-H stretching) shifted to 3380 cm⁻^1^. The characteristic peak at 2862 cm⁻^1^ disappeared, likely because of intramolecular interactions between the rice starch and the pandan pigment through hydrogen bonding [21]. When the rice starch concentration was 3%, the intensity of the broad peak at approximately 3387 cm⁻^1^ in the rice starch–pigment mixture reached a maximum because of the increased number of hydroxyl groups, suggesting enhanced hydrogen bonding between the rice starch and the pandan pigment.

A downwards shift from 3294 to 3289 cm⁻^1^ was observed with the addition of whey protein to the pandan pigment. This shift suggests that whey protein may bind to the pandan pigment through hydrogen bonding. In comparison, the whey protein–pigment mixture shifted from 1649 to 1656 cm⁻^1^ in the amide I (C=O) region and from 1544 to 1541 cm⁻^1^ in the amide II (N-H) region. These changes indicate that the interaction between the pandan pigment and whey protein involves electrostatic interactions [22].

Compared with that of the pandan pigment, the peak intensity of the soy protein isolate–pandan pigment mixture in the 3600–3000 cm⁻^1^ range significantly increased. Furthermore, the C=O stretching vibration peak of the amide I band and the N-H bending vibration absorption peak of the amide II band shifted to some extent. The characteristic peaks at 1739 cm^−1^ and 1615 cm⁻^1^ disappeared in the pandan pigment when the soy protein isolate was bound to the pandan pigment. The amide I and amide II peaks in the whey protein pigment mixture shifted from 1649 cm^−1^ to 1656 cm^−1^ and from 1544 cm^−1^ to 1541 cm^−1^, respectively. These results indicate that interactions, including hydrogen bonding, hydrophobic interactions, and electrostatic interactions, occur between the pandan pigment and soy protein isolate.

In the absence of sucrose, lactose, rice starch, whey protein, and soy protein isolate, the bands at 3389, 2921, and 2847 cm^−1^ increased in intensity, with a relatively small shift, before and after preheating. The peak near 1739 cm^−1^ did not significantly increase or shift after preheating at 65 °C, whereas it markedly increased after preheating at 115 °C.

The different functional groups of the free pandan pigment presented various intense and sharp peaks, which disappeared after it bound to sucrose, lactose, rice starch, whey protein, or soy protein isolate following preheating treatment. With the addition of whey protein and soy protein isolate, the transmittance of the peaks at 2925, 2852, and 1739 cm⁻^1^ decreased, and the peak at 3340 cm⁻^1^ even disappeared.

## 4. Discussion

Natural plant pigments are sensitive to heat, and temperature has a significant effect on the stability of pandan pigment. Higher temperatures lead to faster degradation of the pandan pigment, as observed in this study. The addition of sucrose, lactose, rice starch, whey protein, and soy protein isolate effectively mitigated heat-induced degradation of the pandan pigment. However, the extent of protection varied among these additives, which may be related to the inherent properties of the additives themselves. Compared with sucrose, lactose provides better protection for color stability and thermal stability because of its reducing properties [23]. Rice starch undergoes gelatinization and aging upon heating and cooling, altering its physical structure. Gelatinized starch exposes hydroxyl groups in amylose chains, facilitating interactions with polar organic compounds through hydrogen bonding or hydrophobic interactions and the formation of V-type complexes [24]. Heating significantly increased the surface hydrophobicity of whey protein and soy protein isolate, enhancing their binding with the pandan pigment. Whey protein, which has a greater tryptophan content than soy protein isolate does [25], demonstrated greater stability in the whey protein–pigment complex than in the soy protein isolate–pigment mixture. The concentration of additives significantly affected the stability of the pandan pigment, indicating a concentration-dependent effect. Sucrose provides significant protection at low concentrations but is less effective at high concentrations, which is consistent with its impact on anthocyanins [26].

The influence of the dual factors of temperature and concentration on the color stability of pandan pigment is relatively complex. The L* value and b* value do not change regularly with increasing concentration. This is mainly because the pandan pigments undergo degradation during the heating process. Transition from green to brown. Moreover, this may be related to the highly volatile aromatic compound 2-acetyl-1-pyrroline contained in the pandan pigment. Although 2-acetyl-1-pyrroline itself does not play a direct role in color, it can undergo the Maillard reaction with amino acids, reducing sugars, and other substances in food, resulting in browning [27]. The L* value of the pandan pigment decreases with increasing temperature. For example, the L* value was 17.37 for heating at 65 °C for 15 min, but it decreased to 15.56 for heating at 121 °C for 15 min.This suggests that the pandan pigment was degraded during treatment, resulting in a brownish-yellow color [28]. The addition of sucrose, lactose, whey protein, or soy protein isolate had no significant effect (*p* > 0.05) on the L* values of the pandan pigment. These findings suggest that the formation of sucrose–pandan, lactose–pandan, whey protein–pandan, and soy protein isolate–pandan pigment complexes did not significantly affect the L* values. The addition of rice starch significantly reduced the L* value, possibly because the color of the rice starch obscured the pigment. The L* value decreased with the addition of rice starch and with heating at both 65 °C for 15 min and 95 °C for 15 min. However, at higher temperatures of 115 °C for 15 min and 121 °C for 15 min, the L* value increased. This finding indicates that both the pigment and the rice starch are damaged at these higher temperatures, resulting in a noticeable reduction in color intensity. Pandan pigment contains a significant amount of chlorophyll, imparting a green color and resulting in a lower L* value. The L* value is inversely proportional to the chlorophyll content; as the chlorophyll concentration increases, the brightness decreases [14]. These findings suggest that the addition of sucrose, lactose, rice starch, whey protein, and soy protein isolate help to protect the pandan pigment from heat-induced degradation.

More negative a* values indicate that the color of the pandan pigment is greener [22]. Overall, compared with sucrose and lactose, rice starch provided better color protection for the pandan pigment, and whey protein was more effective than soy protein isolate.

The interaction between the pandan pigment mixture and various concentrations of sucrose, lactose, rice starch, whey protein, and soy protein isolate may explain the delay in the shift of the pandan pigment from green to yellow, contributing to the stabilization of the pandan pigment. At the same time, some studies have shown that the formation of moderately to highly volatile aroma compounds (2-acetyl-1-pyrroline) in pandan requires cooking or the Maillard reaction occurring through contact with food [29]. And previous studies have shown that thermal processing under different temperatures has a positive impact on the aroma profiles and facilitated the formation of ketones, alcohols, and aldehydes [30]. This maintains the uniqueness of the aroma of pandan pigments.

The spectral characteristics of the pandan pigment solutions are shown in Figure 2. Compared with the pandan pigment solution alone, the absorbance of the pandan pigment solution increased in the presence of rice starch, whey protein, and soy protein isolate at different temperatures. It was significantly greater after heating at 65 °C for 15 min and 95 °C for 15 min. This increase was attributed to the interaction between the chromophores in the pandan pigment and the auxochromes (–OH, –NH2, –SH, etc.) in the rice starch, whey protein, and soy protein isolate, resulting in a hyperchromic effect. At 115 °C for 15 min and at 121 °C for 15 min, the absorbance remained stable. Elevated temperatures reduced the absorbance peaks of the pandan pigment because of the decomposition and oxidation of its molecules. These reactions can alter or destroy the chromophores or auxochromes within the pigment molecules. The presence of sucrose and lactose results in instability in the UV absorbance of the pandan pigment, possibly due to the inherent instability of sucrose and lactose, which degrade upon heating. Higher lactose concentrations led to lower absorbance peaks, likely due to the formation of sugar degradation products and subsequent derivatives during heating [31], which may also accelerate pandan pigment degradation. Temperature changes can affect the structure and microenvironment of amino acid residues in whey protein and soy protein isolate, potentially altering their interactions with the pandan pigment [22].

Figure 3 shows the changes in the fluorescence spectra of the pandan pigment mixtures at different temperatures. No concentration-dependent quenching of the pandan pigment fluorophore was observed with the addition of sucrose, lactose, rice starch, whey protein, or soy protein isolate [32]. In contrast, the fluorescence intensity of the pandan pigment increased. This may be a sign that in the presence of sucrose, lactose, rice starch, whey protein, and soy protein isolate molecules, more porphyrin rings of the pandan pigment are exposed, resulting in higher fluorescence intensity [33]. The fluorescence spectra of the pandan pigment and mixtures with sucrose, lactose, rice starch, whey protein, and soy protein isolate overlapped and redshifted after heat treatment (Figure 3). This is due to the high osmotic pressure environment created by sucrose; lactose is destroyed, leading to an increase in water activity [26], and the denaturation of rice starch, whey protein, and soy protein isolates by heating results in the exposure of hydrophobic groups on the molecular surface. This means that the pandan pigment and sucrose, lactose, rice starch, whey protein, and soy protein isolate binding sites in the hydrophobic cavity are destroyed, so the difference in the fluorescence spectrum due to the different numbers of hydrophobic binding sites caused by different concentrations disappears.

There was a significant difference between the pandan pigment and whey protein before and after heating. The fluorescence intensity of the pandan pigment supplemented with whey protein was greater than that of the unamended group before heating. The fluorescence intensity of the pandan pigment supplemented with whey protein was lower than that of the untreated group after heating. The reason may be that the pyrrole ring of the pandan pigment becomes bound to the hydrophobic residues of whey protein, and pandan pigment self-aggregation and whey protein self-aggregation are preferable to the binding of the pandan pigment and whey protein. Heat also destroys the binding sites of pandan pigment self-aggregation and whey protein self-aggregation [34]. This leads to increased interaction of pyrrole rings with the whey protein.

The fluorescence intensities of the pandan pigment and sucrose, lactose, rice starch, and soy protein isolates increased after heating, indicating that the pandan pigment was bound to sucrose, lactose, rice starch, and soy protein isolate through phytyl. Heating destroyed the binding sites of pandan pigment self-aggregation, increasing the number of porphyrin rings exposed, and thus, increasing the fluorescence intensity.

For all the pandan pigment and the sucrose, lactose, rice starch, whey protein, and soy protein isolate, the characteristic hydroxyl peak (O-H stretching) was observed at approximately 3400 cm^−1^. Compared with the peak intensities for the pure pandan pigment, the increase in and broadening of the peak at approximately 3340 cm^−1^, corresponding to the intermolecular hydrogen bonds and O-H stretching modes in the mixtures, indicated the presence of hydrogen bonds between the pandan pigment and the sucrose, lactose, rice starch, whey protein, and soy protein isolate. We observed a broad absorption band in the 3000–3600 cm^−1^ region, which reveals the presence of a significant proportion of intermolecularly hydrogen-bonded hydroxyl groups before heating. After heating, this broad band gradually narrowed due to the increased thermal motion of the molecules and the weakening of intermolecular forces, which allowed the hydroxyl groups to vibrate more freely and in a more orderly way.

It can be observed that the intensity of the amide I and II bands in the heated pandan pigment-whey protein mixture and pandan pigment-soy protein isolate mixture decreases, or even disappears, indicating a reduction in the content of α-helical structures in the proteins.These changes in the intensities of the amide I and II bands were due to the interactions among whey protein, soy protein isolate, and pandan pigment via hydrogen bonding and hydrophobic attractions [19].

The interaction between whey protein and pigments basically disappeared after heating at 115 °C for 15 min, and the interaction between soy protein isolate and pigments was destroyed after heating at 95 °C for 15 min. This may be due to the thermal denaturation of the proteins themselves. The spectra of the complex of the pandan pigment with sucrose, lactose, and rice starch before and after preheating treatment show no obvious difference in the characteristic absorption peaks. These findings indicate that preheating treatment affects the interaction between the pandan pigment and whey protein or soy protein isolate. The use of the pandan pigment improved the ratio of 1077/1022 cm^−1^, indicating that it increased the ordered structure of the rice starch and promoted the rearrangement of starch molecules. This may be because the pandan pigment can form a V-type compact mixture with rice starch by being encapsulated in amylose. This also explains why the combination of the pandan pigment and rice starch after heating is more stable than the combination of sucrose, lactose, whey protein, and soy protein isolate.

## 5. Conclusions

Through thermal and color stability testing, sucrose, lactose, rice starch, whey protein, and soy protein isolate were found to enhance the stability of the pandan pigment. The interaction between the pandan pigment and these substances, both before and after preheating, was extensively studied via spectroscopic techniques. The results indicate that both native and preheated pandan pigment predominantly bind to sucrose, lactose, and rice starch through hydrophobic forces and hydrogen bonds. In contrast, the binding of the pandan pigment to whey protein and soy protein isolate was driven mainly by hydrogen bonds, hydrophobic interactions, and electrostatic forces. Different preheating temperatures affected the binding affinity and structural conformations of these substances to varying extents. Preheating had a minimal effect on the microenvironment between the pandan pigment and sucrose, lactose, or rice starch, with no significant changes observed in the binding characteristics or interactions. However, the interaction between the pandan pigment and whey protein or soy protein isolate decreased with increasing temperature, as evidenced by the reduced binding affinity. After heating, the pandan pigment was more stable with rice starch than with sucrose, lactose, whey protein, or soy protein isolate. Notably, rice starch still has protective effects on the pigment when heated at 115 °C for 15 min.

## Figures and Tables

**Table 1:** Effects of different concentrations of sucrose, lactose, and rice starch at various heating temperatures on the color stability of the pandan pigment.

		65 °C			95 °C			115 °C			121 °C		
		Sucrose	Lactose	Rice Starch	Sucrose	Lactose	Rice Starch	Sucrose	Lactose	Rice Starch	Sucrose	Lactose	Rice Starch
L*	0%	17.37 ± 0.01	17.37 ± 0.0	17.37 ± 0.01	16.86 ± 0.03	16.86 ± 0.03	16.86 ± 0.03	15.3 ± 0.01	15.3 ± 0.01	15.3 ± 0.01	15.56 ± 0.01	15.56 ± 0.01	15.56 ± 0.01
	1%	16.56 ± 0.01 ***	16.24 ± 0 *	14.08 ± 0 *	15.95 ± 0.02 **	16.42 ± 0.0 *	17.06 ± 0.0 ^ns^	16.72 ± 0.02 **	15.88 ± 0.0 ***	15.53 ± 0.0 ^ns^	15.81 ± 0.11 ^ns^	15.57 ± 0 ^ns^	17.2 ± 0.01 ***
	3%	16.50 ± 0.01 **	16.74 ± 0.5 ^ns^	13.35 ± 0.02 **	16.63 ± 0.04 ^ns^	16.09 ± 0 *	15.66 ± 0.0 *	16.18 ± 0.04 *	15.14 ± 0.0 **	15.36 ± 0.0 *	16.31 ± 0.04 *	15.61 ± 0.0 *	18.41 ± 0.0 ***
	5%	16.65 ± 0.16 ^ns^	15.99 ± 0.0 *	13.67 ± 0 *	16.24 ± 0.04 *	16.66 ± 0.0 ^ns^	16.06 ± 0.0 *	15.59 ± 0.21 ^ns^	15.05 ± 0.0 *	13.45 ± 0.0 ***	15.37 ± 0.00 *	15.75 ± 0 ***	17.42 ± 0.0 **
	7%	16.23 ± 0.01 **	16.48 ± 0.1 ^ns^	14.32 ± 0.03 *	16.83 ± 0.06 ^ns^	16.67 ± 0.0 ^ns^	16 ± 0.01 *	15.26 ± 0.01 ^ns^	16.53 ± 0.0 ***	14.43 ± 0.0 *	15.33 ± 0.01 **	16.75 ± 0.0 *	17.66 ± 0.01 ***
a*	0%	−2.69 ± 0.02	−2.69 ± 0.02	−2.69 ± 0.02	−2.73 ± 0.02	−2.73 ± 0.02	−2.73 ± 0.02	−1.18 ± 0.02	−1.18 ± 0.02	−1.18 ± 0.02	−1.05 ± 0.00	−1.05 ± 0	−1.05 ± 0
	1%	−2.15 ± 0.01 ^ns^	−3.48 ± 0.07 ^ns^	−3.76 ± 0 *	−2.57 ± 0.03 ^ns^	−2.84 ± 0.04 ^ns^	−2.82 ± 0.01 ^ns^	−1.60 ± 0.00 *	−1.43 ± 0.02 *	−1.64 ± 0.01 *	−1.28 ± 0.02 ^ns^	−1.36 ± 0.02 ^ns^	−1.48 ± 0.01 *
	3%	−2.14 ± 0.01 *	−3.99 ± 0 *	−4.22 ± 0 *	−2.32 ± 0.01 *	−2.95 ± 0.04 ^ns^	−3.1 ± 0.02 *	−1.41 ± 0.00 ^ns^	−1.76 ± 0.02 **	−1.84 ± 0.01 **	−1.14 ± 0.00 ***	−1.41 ± 0.01 *	−1.69 ± 0.01 *
	5%	−2.12 ± 0.00 *	−3.48 ± 0.02 ***	−4.65 ± 0.06 **	−2.25 ± 0.02 **	−2.88 ± 0.02 ^ns^	−3.23 ± 0.04 *	−1.4 ± 0.00 ^ns^	−1.33 ± 0.01 ^ns^	−2.91 ± 0.01 **	−1.13 ± 0.03 ^ns^	−0.98 ± 0 ***	−2.04 ± 0 ***
	7%	−2.05 ± 0.07 ^ns^	−3.22 ± 0.02 **	−4.79 ± 0.04 **	−2.32 ± 0.01 *	−2.87 ± 0.02 ^ns^	−3.39 ± 0.04 *	−1.22 ± 0.01 ^ns^	−1.04 ± 0.01 ^ns^	−3.38 ± 0 *	−0.85 ± 0.03 ^ns^	−0.62 ± 0 ***	−2.34 ± 0.01 *
b*	0%	15.11 ± 0.17	15.11 ± 0.17	15.11 ± 0.17	14.85 ± 0.19	14.85 ± 0.19	14.85 ± 0.19	13.65 ± 0.01	13.65 ± 0.01	13.65 ± 0.01	13.81 ± 0.06	13.81 ± 0.06	13.81 ± 0.06
	1%	14.67 ± 0.05 ^ns^	15.16 ± 0.0 ^ns^	12.73 ± 0.23 *	14.05 ± 0.04 ^ns^	14.41 ± 0 ^ns^	11.07 ± 0.03 *	14.84 ± 0.1 ^ns^	15.16 ± 0.0 ***	10.91 ± 0.0 **	13.97 ± 0.28 ^ns^	14.28 ± 0.0 ^ns^	9.85 ± 0.02 **
	3%	14.37 ± 0.06 ^ns^	15.95 ± 0.2 ^ns^	11.83 ± 0.02 *	13.84 ± 0.06 ^ns^	14.45 ± 0.0 ^ns^	10.98 ± 0 *	15.25 ± 0.03 **	13.53 ± 0.0 ^ns^	11.03 ± 0.0 **	14.08 ± 0.23 ^ns^	13.7 ± 0.05 ^ns^	8.64 ± 0.02 **
	5%	14.55 ± 0.18 ^ns^	15.61 ± 0.1 ^ns^	12.56 ± 0.04 ^ns^	14.6 ± 0.06 ^ns^	13.71 ± 0 ^ns^	11.01 ± 0.01 *	14.35 ± 0.26 ^ns^	11.38 ± 0.0 **	11.86 ± 0.0 *	13.73 ± 0.01 ^ns^	11.61 ± 0.0 *	9.4 ± 0.01 *
	7%	15.11 ± 0.01 ^ns^	16.6 ± 0.13 *	13.17 ± 0.01 ^ns^	14.65 ± 0.11 ^ns^	13.14 ± 0 ^ns^	10.95 ± 0.01 *	14.42 ± 0.09 ^ns^	10.62 ± 0 *	12.73 ± 0.0 ***	13.59 ± 0.03 ^ns^	9.29 ± 0.01 *	10.09 ± 0.0 **

Note: L* (brightness), a* (greenness–redness), and b* (blueness–yellowness). “ns” represents not significant, “*”, “**”, and “***” represent significance levels of 0.05, 0.01, and 0.001, respectively. The values are the means ± standard deviations. The significant difference refers to comparing the same substance at different temperatures. The same applies below.

**Table 2:** Effects of different concentrations of whey protein and soy protein isolate at various heating temperatures on the color stability of the pandan pigment.

		65 °C		95 °C		115 °C		121 °C	
		Whey Protein	Soy Protein isolate	Whey Protein	Soy Protein Isolate	Whey Protein	Soy Protein Isolate	Whey Protein	Soy Protein Isolate
L*	0%	17.37 ± 0.01	17.37 ± 0.01	16.86 ± 0.03	16.86 ± 0.03	15.30 ± 0.01	15.30 ± 0.01	15.56 ± 0.01	15.56 ± 0.01
	2%	16.05 ± 0.07 *	16.15 ± 0.01 **	15.17 ± 0.08 *	16.12 ± 0.00 *	15.16 ± 0.01 **	15.13 ± 0.02 ^ns^	14.4 ± 0.01 ***	15.61 ± 0.01 ^ns^
	4%	14.84 ± 0.00 *	16.27 ± 0.00 *	15.25 ± 0.01 **	15.65 ± 0.01 *	14.8 ± 0.00 *	15.43 ± 0.01 **	14.46 ± 0.01 **	15.39 ± 0.01 *
	6%	15.2 ± 0.01 ***	16.4 ± 0.01 ***	14.46 ± 0.00 *	15.58 ± 0.01 **	14.27 ± 0.01 ***	15.95 ± 0.06 ^ns^	14.00 ± 0.02 **	15.44 ± 0.00 ^ns^
	8%	15.21 ± 0.01 **	15.37 ± 0.04 *	14.94 ± 0.01 *	16.61 ± 0.06 ^ns^	13.8 ± 0.02 **	15.27 ± 0.00 ^ns^	14.12 ± 0.01 **	15.37 ± 0.01 **
a*	0%	−2.69 ± 0.02	−2.69 ± 0.02	−2.73 ± 0.02	−2.73 ± 0.02	−1.18 ± 0.02	−1.18 ± 0.02	−1.05 ± 0.00	−1.05 ± 0.00
	2%	−4.27 ± 0.01 **	−3.82 ± 0.01 **	−3.96 ± 0.00 *	−3.74 ± 0.05 *	−2.98 ± 0.01 **	−3.17 ± 0.00 *	−2.58 ± 0.01 *	−2.88 ± 0.00 ***
	4%	−4.63 ± 0.04 **	−4.05 ± 0.05 **	−4.37 ± 0.01 **	−3.94 ± 0.00 *	−3.97 ± 0.01 ***	−3.42 ± 0.03 ***	−3.65 ± 0.01 *	−3.68 ± 0.00 ***
	6%	−4.76 ± 0.01 **	−4.14 ± 0.03 ***	−4.86 ± 0.03 ***	−4.13 ± 0.03 ***	−4.29 ± 0.01 **	−4.05 ± 0.00 *	−3.72 ± 0.01 *	−3.84 ± 0.04 *
	8%	−4.89 ± 0.01 **	−4.21 ± 0.01 ***	−4.93 ± 0.01 ***	−4.47 ± 0.00 *	−4.34 ± 0.04 **	−4.34 ± 0.01 **	−3.86 ± 0.00 ***	−3.78 ± 0.01 *
b*	0%	15.11 ± 0.17	15.11 ± 0.17	14.85 ± 0.19	14.85 ± 0.19	13.65 ± 0.01	13.65 ± 0.01	13.81 ± 0.06	13.81 ± 0.06
	2%	15.64 ± 0.08 ^ns^	14.98 ± 0.08 ^ns^	13.75 ± 0.08 ^ns^	15.55 ± 0.04 ^ns^	14.64 ± 0.01 **	14.97 ± 0.09 ^ns^	12.79 ± 0.03 *	15.69 ± 0.01 *
	4%	14.43 ± 0.01 ^ns^	15.33 ± 0.06 ^ns^	14.66 ± 0.01 ^ns^	14.48 ± 0.01 ^ns^	15.04 ± 0.03 **	15.24 ± 0.08 *	13.76 ± 0.04 ^ns^	14.78 ± 0.04 *
	6%	15.29 ± 0.02 ^ns^	15.32 ± 0.01 ^ns^	13.82 ± 0.04 ^ns^	14.68 ± 0.04 ^ns^	14.15 ± 0.05 ^ns^	16.34 ± 0.08 *	13.29 ± 0.04 *	15.71 ± 0.03 **
	8%	15.27 ± 0.08 ^ns^	14.47 ± 0.35 ^ns^	14.42 ± 0.01 ^ns^	16.18 ± 0.06 ^ns^	14.09 ± 0.05 ^ns^	15.78 ± 0.01 ***	14.04 ± 0.01 ^ns^	15.65 ± 0.04 **

Note: L* (brightness), a* (greenness–redness), and b* (blueness–yellowness) “ns” represents not significant, “*”, “**”, and “***” represent significance levels of 0.05,0.01, and 0.001, respectively. The values are the means ± standard deviations. The significant difference refers to comparing the same substance at different temperatures.

## Data Availability

The original contributions presented in the study are included in the article, further inquiries can be directed to the corresponding authors.
